# Dimethyl fumarate (DMF) vs. monoethyl fumarate (MEF) salts for the treatment of plaque psoriasis: a review of clinical data

**DOI:** 10.1007/s00403-018-1825-9

**Published:** 2018-03-24

**Authors:** Lilla Landeck, Khusru Asadullah, Adriana Amasuno, Ignasi Pau-Charles, Ulrich Mrowietz

**Affiliations:** 1 Department of Dermatology, Ernst von Bergmann General Hospital, Teaching Hospital Charité, Humboldt University, Charlottenstrasse 72, 14467 Potsdam, Germany; 2 Department of Dermatology, Venerology and Allergology, Charité Universitätsmedizin Berlin, Berlin, Germany; 3 Almirall S.A., Barcelona, Spain; 4 Psoriasis-Center at the Department of Dermatology, University Medical Center Schleswig-Holstein, Campus Kiel, Germany

**Keywords:** Dimethyl fumarate, DMF, Monoethyl fumarate, MEF, Psoriasis, Fumaric acid esters

## Abstract

Fumarates (fumaric acid esters, FAEs) are orally administered systemic agents used for the treatment of psoriasis and multiple sclerosis. In 1994, a proprietary combination of FAEs was licensed for psoriasis by the German Drug Administration for use within Germany. Since then, fumarates have been established as one of the most commonly used treatments for moderate-to-severe psoriasis in Germany and other countries. The licensed FAE formulation contains dimethyl fumarate (DMF), as well as calcium, zinc, and magnesium salts of monoethyl fumarate (MEF). While the clinical efficacy of this FAE mixture is well established, the combination of esters on which it is based, and its dosing regimen, was determined empirically. Since the mid-1990s, the modes of action and contribution of the different FAEs to their overall therapeutic effect in psoriasis, as well as their adverse event profile, have been investigated in more detail. In this article, the available clinical data for DMF are reviewed and compared with data for the other FAEs. The current evidence substantiates that DMF is the main active compound, via its metabolic transformation to monomethyl fumarate (MMF). A recent phase III randomized and placebo-controlled trial including more than 700 patients demonstrated therapeutic equivalence when comparing the licensed FAE combination with DMF alone, in terms of psoriasis clearance according to the Psoriasis Area and Severity Index (PASI) and Physician’s Global Assessment (PGA). Thus, DMF as monotherapy for the treatment of psoriasis is as efficacious as in combination with MEF, making the addition of such fumarate derivatives unnecessary.

## Introduction

Psoriasis is a chronic inflammatory skin disease with various subtypes, of which chronic plaque psoriasis is the most common [21]. While mild disease is predominantly treated with topical agents, therapy of moderate-to-severe disease requires systemic treatment.

### The role of fumarates in the therapy of psoriasis

Current guidelines provide an overview of appropriate systemic therapy for moderate-to-severe plaque psoriasis with conventional and biological agents. Conventional oral systemic agents include acitretin, ciclosporin, methotrexate, and fumaric acid esters (FAEs; fumarates) [33]. FAEs are ester derivatives of FA (Table 1). Major derivatives of interest for oral therapy are dimethyl fumarate (DMF) and monoethyl fumarate (MEF) and its salts.

**Table 1:** Chemical structure and basic properties of free FA and the FAEs: DMF, MEF and three salts of MEF (Adapted from Brennan et al. [10])

Compound	Molecular formula	Molecular weight	Melting points (ºC)	Water solubility (ng/mL)	Acid dissociation constant (pKa)	Molecular structure
DMF	C_6_H_8_O_4_	144.13	102	1	No ionizable protons	
FA	C_4_H_4_O_4_	116.07				
MEF	C_6_H_8_O_4_	144.13				
MEF-Ca	C_12_H_14_CaO_8_	326.31	285	294	3.3	
MEF-Zn	C_12_H_14_O_8_Zn	351.62	300	300	3.3	
MEF-Mg	C_12_H_14_MgO_8_	310.54	169	826	3.3	

Schweckendiek was the first to propose in 1959 that psoriasis was caused by a disturbance involving FA in the citric acid cycle [5, 39]. Subsequently, Kiehl and Ionescu [18] described a defective purine nucleotide synthesis pathway in patients with psoriasis. These authors noted a correlation between increased adenosine triphosphate (ATP) levels in blood cells after administration of FA and FAEs and clearance of skin lesions. Although FA deficiency is not known as a cause of disease in humans, a proprietary mixture of FAEs (Fumaderm®, Biogen Idec), with an empirically determined dosing schedule, became established as a first-line systemic therapy for moderate-to-severe psoriasis in Germany, with a reported efficacy comparable to other systemic agents, such as methotrexate [41].

This particular FAE combination was first registered in Germany in 1994, and the current formulation is an enteric-coated tablet, available in two dosages (Fumaderm® initial and Fumaderm®), which contain DMF 30 mg or 120 mg and three salts of MEF, MEF-Ca (67 mg or 87 mg), MEF-Zn (both 3 mg) and MEF-Mg (both 5 mg), respectively [8]. Psorinovo® is a pharmacy-compounded DMF-only formulation (enteric-coated tablets, GMP Apotheek Mierlo-Hout) that is used to treat psoriasis in the Netherlands [35], where the local psoriasis treatment guidelines recommend FAEs as an induction treatment on moderate-to-severe disease [42]. In addition to psoriasis, DMF is used as the first-line therapy for relapsing–remitting multiple sclerosis [4, 22, 23, 25]. A delayed-release oral formulation of DMF, Tecfidera® (120 and 240 mg), has been approved for this indication in Europe [15] and the US [9].

The safety profile of fumarates has been established through decades of clinical use. Adverse events (AEs) may affect up to two-thirds of patients [5, 38]. However, AEs are usually mild, and most commonly include gastrointestinal symptoms such as diarrhoea, stomach ache, cramps, increased frequency of stools, nausea, and vomiting [5, 26, 38]. Formulation research to address this problem has led to the delayed-release formulation of the currently licensed FAE combination [8]. Other common AEs include flush, leukocytopenia and lymphopenia [2, 17], and reversible peripheral eosinophilia. In fact, all FAE-containing products approved for treatment of psoriasis require periodic blood monitoring and, depending on the severity of lymphopenia, incorporate their own treatment discontinuation guidelines as part of their prescribing information, to prevent opportunistic infections [8]. A few cases of progressive multifocal leukoencephalopathy (PML), a rare, opportunistic viral infection of the central nervous system characterized by progressive inflammation and damage to the brain, have been reported with the use of FAEs in patients with long-standing and pronounced lymphopenia. In all cases, patients had not been appropriately monitored. PML is indeed a clinically relevant risk; however, the risk is believed to be minimal, should appropriate and periodic blood monitoring be carried out in FAE-treated patients [30].

In terms of their metabolism, FAEs are completely absorbed in the small intestine [28]. DMF has a half-life of approximately 12 min and is hydrolyzed to monomethyl fumarate (MMF; also known as methyl hydrogen fumarate, MHF) which has a half-life of 36 h [28]. MMF reaches peak plasma concentrations after 5–6 h, is metabolized via the citric acid cycle to fumaric acid, water and carbon dioxide, and is excreted mainly through the breath [28]. DMF is considered to act as a prodrug of MMF. DMF has been the primary orally administered fumarate of interest in most preclinical studies.

Much of the available data regarding the mode of action of FAEs in psoriasis have been obtained using the licensed FAE combination; however, the relative contributions of each FAE component to the therapeutic activity remained unclear. Despite its widespread acceptance in Germany, as an empirical mixture of different FAEs, this FAE combination remains unapproved for psoriasis elsewhere [26]. Current European S3 guidelines recommend FAEs for the short- and long-term treatment of moderate-to-severe plaque psoriasis [33]. FAEs are also included in US guidelines, but with the caveat that they are not registered in that country [24]. The Cochrane Collaboration stated that while FAEs are superior to placebo for the treatment of psoriasis, there was still a need for more robust clinical trials and long-term safety data [5]. A recent phase III, double-blind, placebo-controlled study is a recent trial fulfilling such needs [32].

Altogether, an improved understanding of the relative contribution of each individual FAE compound toward their overall therapeutic effect in psoriasis is desirable. As a general principle, single-compound preparations (i.e. medicines with just one active pharmaceutical ingredient) are preferable over ‘combination products’ containing different active agents. The presence of compounds in a product that is not necessary for the therapeutic effect should be critically discussed, because of possible side effects.

The aim of this review is to evaluate the available clinical data regarding the effects of individual FAEs, in particular DMF and MEF, to describe the relative contribution of these compounds to the effectiveness of the approved FA mixture in the management of psoriasis.

## Method

All relevant scientific publications relating to DMF and other FAEs were identified through a comprehensive search of the scientific literature. The databases searched were: PubMed/Medline and EMBASE (which includes the Cochrane library). Our search strategy included general chemical names for DMF and MEF and was not limited by any specific time period; thus, the entire database to January 23, 2018 was covered. To capture as many relevant hits as possible, the following search terms were used: (“dimethyl fumarate”) OR (“dimethylfumarate”) OR (“DMF”) OR (“ethyl fumarate”[NM]) OR (“monoethyl fumarate”) OR (“monoethylfumarate”) OR (“MEF”) OR (“monomethyl fumarate”) OR (“monomethylfumarate”) OR (“MMF”) OR (“Fumaric acid ester”) OR (“Fumaric acid ethyl ester”) OR (“Fumaric acid monoethyl ester”) OR (“fumaric acid monomethyl ester”). The search was narrowed down further by adding the term “psoriasis”. All retrieved citations were screened and the most relevant were selected for inclusion in this review. In addition, supplementary references were identified by searching through the bibliography cited within the retrieved publications.

## Results

### Clinical studies of FAEs in psoriasis

The efficacy of the FAEs in psoriasis was initially determined empirically. Many of the early clinical studies were not placebo controlled and without a comparator arm [1, 7, 19, 27, 37]. Nieboer et al. were first to explore the differences between FAEs systematically [37]. In a series of five open/controlled studies in small numbers of patients, they verified MEF monotherapy as being not superior to placebo as assessed by a Psoriasis Severity Score (PSS). In contrast, DMF monotherapy was superior to placebo [37]. Results of these studies are summarized in Table 2. DMF 240 mg daily significantly improved PSS scores over 6 weeks in study III, and in the long-term continuation study V (4–9 months) it was shown that DMF monotherapy led to moderate or considerable improvement in 22 and 33% of patients, respectively (Table 2). In contrast, 240 mg daily of MEF-Na had no benefit vs. placebo in study II. While a higher Na-MEF dose (720 vs. 240 mg; study IV) was associated with greater improvements in skin scaling and itching, there was no relevant difference between the two arms in the number of patients with considerable (> 50%) improvement (*n* = 3 in both groups).

**Table 2:** Comparative studies by Nieboer et al. [37] examining fumaric acid esters (FAEs) in patients with stable nummular or plaque psoriasis (≥ 10% of body surface area)

Study number (all published by Nieboer et al. [37])		Design and duration	Regimen	No. of patients	Outcome measures (all studies)	Main efficacy findings	Main safety findings
Study I		nb 1–32 months	FACT	36	PSS; haematological and biochemical parameters	64 and 44% of patients showed > 50 and > 90% improvement Itching and scaling improved after 1 month	One patient stopped due to serious GI AEs
Study II		db 4 months	Na-MEF 240 mg vs. Placebo	19 vs. 19		No difference between Na-MEF and placebo; greater itching score drop with Na-MEF	None reported
Study III		db 4 months	DMF 240 mg vs. Placebo	22 vs. 20		Significant differences between DMF and placebo (*p* < 0.01) within 6 wk: PSS ↓ to 60% for DMF vs. ↑ to 105% with placebo	27% of patients stopped due to serious GI AEs during the first 2 weeks
Study IV	Dose finding 3 months		Na-MEF 720 mg vs. 240 mg	10 vs. 10		Na-MEF doses equivalent for no. of improved patients; significant differences (*p* < 0.05) between final itching and scaling scores of scaling	None reported
Study V	nb 4–9 months (continuation study)		DMF 60–240 mg	56		22% moderate improvement; 33% > 50% improvement	20% of patients discontinued due to serious GI AEs

*AE* adverse event, *db* double-blind, *DMF* dimethylfumarate, *FACT* fumaric acid compound therapy (oral DMF + MEF), *GI* gastrointestinal, *Na-MEF* sodium monoethylfumarate, *nb* nonblind, *PSS* psoriasis severity score, *wk* week(s)

Gastrointestinal complaints led to discontinuation in 20–27% of patients receiving DMF treatment [37]. Mild and reversible liver and kidney function abnormalities were noted with MEF 720 mg/day and DMF 240 mg/day. Almost 60% of patients treated with DMF alone experienced lymphopenia (mean % lymphocyte counts in peripheral blood in these patients dropped from 21.9 to 11.5%). However, this resolved approximately 6 months after treatment discontinuation. Interestingly, there was a statistically significant correlation between a ≥ 50% PSS improvement and lymphopenia (*p* < 0.01) during DMF therapy [37].

In 1990, a subsequent double-blind study by the same group (comparing DMF and a DMF plus MEF salts mixture) showed > 50% PSS improvement in 55% of 22 DMF and 80% of 23 DMF plus MEF patients after 4 months’ treatment. At the same time, a higher rate of treatment discontinuations in the combination arm (47 vs. 17%) was observed [36]. There were no significant differences between DMF and the DMF/MEF combination regarding the total PSS or individual parameters (e.g. extent of lesions, induration, scaling, redness and itching). Although treatment effects were seen a few days earlier with the DMF/MEF combination (*n* = 15) than with DMF alone (*n* = 18), all final scores after 4 months were not statistically different between the groups [36]. Overall, the results from these trials suggested that DMF is the active moiety in this formulation, and that the addition of other compounds may increase the risk of AEs without additional therapeutic benefit. On the basis of the above-mentioned studies, the FAE combination was approved in Germany.

Further evidence for the efficacy of DMF monotherapy was provided by Mrowietz et al. [31] in a randomized, double-blind and placebo-controlled study that fulfilled conditions for inclusion in the Cochrane review of Atwan et al. [5]. Randomized patients received an oral formulation of DMF 120 mg encapsulated as enteric-coated granules (microtablets). Patients with moderate-to-severe disease received 240 mg titrated over the first 7 days (*n* = 105) or placebo (*n* = 70) three times a day for 16 weeks. The median Psoriasis Area and Severity Index (PASI) reduction achieved with DMF was significantly higher than with placebo (67.8 vs. 10.2%; *p* < 0.001), and there was a 47% improvement in the Skindex-29 quality-of-life score when compared with placebo in the active treatment group. The most common AEs with DMF were gastrointestinal complaints (58 vs. 23% with placebo) and flush (42 vs. 9%). Gastrointestinal complaints were generally of mild or moderate severity and not treatment limiting [5, 31].

The encapsulated microtablet formulation of DMF was also investigated in a phase II multicentre, double-blind, placebo-controlled, dose-ranging study in Poland [20]. This trial included a 24-week, open-label safety-extension phase, and involved patients with chronic plaque, exanthematic guttate, erythrodermic, palmoplantar or pustular psoriasis of at least 1 year duration and a baseline PASI of 16–24. In the double-blind phase, 144 patients were randomized to placebo or to DMF 120, 360 or 720 mg daily for 12 weeks. A dose-related improvement with DMF vs. placebo was noted by 2 weeks, and the DMF formulation was well tolerated, with gastrointestinal complaints reported infrequently. The open-label extension phase subsequently enrolled 108 patients [20].

A recent study in 28 psoriasis patients treated with DMF (for a minimum of 6 weeks before inclusion in the study) and 32 healthy controls has also reported significantly reduced levels of the faecal *S. cerevisiae* abundance in psoriasis patients vs. controls (*p* < 0.001). Following 6–9 weeks of treatment with DMF, levels of *S. cerevisiae* were restored to levels similar to those of the healthy controls (*p* = 0.233). Moreover, gastrointestinal side effects as reported in DMF-treated patients were correlated with enhanced *S. cerevisiae* levels. As *S. cerevisiae* is known to have beneficial immunomodulatory properties, this may offer a previously uninvestigated means by which DMF elicits its anti-psoriatic effects, i.e. via restoration of an anti-inflammatory microbiome [14].

### Clinical head-to-head comparisons of DMF and FAE combination

In 1992, Kolbach and Nieboer [19] examined the efficacy and safety of DMF monotherapy in comparison with the FAE combination over 4 months in a prospective, randomized study in 196 patients with nummular or plaque-type psoriasis (Table 3) [19]. This study had a notable influence on the direction of FAE development in psoriasis over the next two decades. The patients in the DMF group were treated with capsules filled with a semienteric-coated DMF granulate, and in the combination group a DMF/MEF mixture (Fumaderm®) was used. Topical treatment was permitted and consisted of bland cream/ointment or a mild corticosteroid. Patients were evaluated after 3–6, 6–12, 12–18 and 18–24 months. A simplified form of the PSS was used, with > 75% improvement classified as ‘sufficient’, less extensive improvement as ‘deterioration’, and exacerbation as ‘insufficient’.

**Table 3:** Randomized comparisons of dimethyl fumarate (DMF) vs. FAE combinations in clinical studies

Study	Design	Regimen	No. of patients	Diagnosis	Main outcome measures	Main efficacy findings	Main safety findings
Kolbach and Nieboer [19]	r	DMF (60–240 mg/day) × 24 mo	129	Nummular and plaque psoriasis (≥ 10% of BSA) n.b. Excluded: generalized pustular psoriasis	Simplified PSS	> 75% improvement in 18–32% of patients Yr 2 dropout rate = 84%	Frequent GI AEs during the first 6 mo Lymphopenia seen by 3 mo; frequent (85%) by 24 mo
		FAEs (containing DMF 120 mg, up to 4 × daily) × 24 mo	67			> 75% improvement in 46–51% of patients Yr 2 dropout rate = 45%	
Mrowietz et al. [32]	r, db, mc, pc	DMF up to 720 mg tid	279	Moderate-to-severe chronic plaque psoriasis; ≥ 12 mo duration + PASI > 10 and > 10% BSA n.b. Excluded: guttate, erythrodermic or pustular psoriasis	PASI 75; PGA; BSA	PASI 75 wk 16 = 37.5%*† PGA ‘clear’ or ‘almost clear’ = 33%* BSA improvement wk 16 = 13.2 (*p* < 0.001 vs. placebo)	TEAEs = 83.9% Lymphopenia = 10.0% GI TEAEs = 62.7%
		FAEs (up to 720 mg of DMF tid)	283			PASI 75 wk 16 = 40.3%* PGA ‘clear’ or ‘almost clear’ = 37.4%* BSA improvement wk 16 = 11.3 (*p* < 0.001 vs. placebo)	TEAEs = 84.1% Lymphopenia = 10.6% GI TEAEs = 63.3%
		Placebo	137			PASI 75 wk 16 = 15% PGA ‘clear’ or ‘almost clear’ = 13% BSA improvement = 4.9	TEAEs = 59.9% Lymphopenia = 0%

*BSA* body surface area involvement, *db* double-blind, *DMF* dimethylfumarate, *GI* gastrointestinal, *mc* multicentre, *mo* month(s), *PASI 75* Psoriasis Area and Severity Index 75% improvement, *pc* placebo controlled, *PGA* Physician’s Global Assessment, *PSS* Psoriasis Severity Score, *r* randomized, *TEAE* treatment-emergent adverse event, *tid* 3 times daily, *yr* year(s)

*p < 0.001 for superiority vs. placebo

^†^*p* < 0.001 for noninferiority vs. FAEs

This trial showed apparent superiority of the FAE mixture over DMF monotherapy. After 24 months, 55% of patients continued on the FAE mixture therapy, while only 16% remained on DMF (*p* < 0.05). Approximately, 50% of recipients of the FAE mixture showed ‘sufficient’ results over the entire study whereas in the DMF group the proportion of ‘sufficient’ responders declined from 32 to 18% over the 24-month study timeframe. Among responders, the first signs of improvement were generally seen after 3 weeks, with resolution of lesions in the following weeks. Decreased arthralgia severity was noted in 27% of patients treated with DMF and in 50% of patients treated with the FAE mixture. The most prominent reasons for discontinuation of therapy were an ‘insufficient’ result in the DMF group (36%) and AEs in the FAE mixture group (18%). As the authors concluded from their data that the FAE mixture was significantly more effective than DMF alone in the management of psoriasis, subsequent studies tended to focus on combination treatment with FAEs. However, this trial was subject to a number of notable shortcomings. Details of the randomization scheme used were not given, and patients in the FAE mixture group received the equivalent of twice as much DMF as those on DMF monotherapy (up to 480 mg of DMF daily vs. up to 240 mg; Table 3). In addition, a more prolonged dose titration regimen was used in the FAE mixture group (7 weeks involving two tablet strengths, in comparison to 4 weeks with a single strength of DMF), patient demographics were not reported, and dropout rates after 2 years were very high in both arms. Most importantly, the galenical formulation of the FAE mixture differed substantially from the capsules containing a semienteric-coated DMF granulate. As there was no pharmacologic profiling of both formulations, the treatment groups cannot be compared with each other.

More recently, a head-to-head comparator trial has shown a similar clinical response to the licensed FAE mixture (Fumaderm®) and DMF alone, when compared against placebo [32]. This was a phase III randomized and placebo-controlled trial in which patients were assigned to treatment with a new formulation of DMF (LAS41008), the FAE mixture or placebo in a 2:2:1 ratio for 16 weeks in four European countries (Table 3). Patients were followed up for up to 12 months after treatment discontinuation. Notably, uptitration of DMF dosage was the same for DMF and the FAE mixture in this study, and the maximum allowed daily dose was the same in both active therapy groups (720 mg of DMF).

Assessments were based on the European Medicines Agency’s clinical investigation guidance, and consisted of the PASI, Physician’s Global Assessment (PGA; six-point scale) and body surface area involvement (BSA). Primary efficacy endpoints were the proportion of patients achieving PASI 75 (≥ 75% improvement vs. baseline, considered clinically meaningful) and the proportion achieving PGA of 0 or 1 (‘clear’ or ‘almost clear’) at week 16. Other endpoints included BSA, PASI 50 and PASI 90, and primary efficacy endpoints reported at 3 and 8 weeks.

**Fig. 1:**
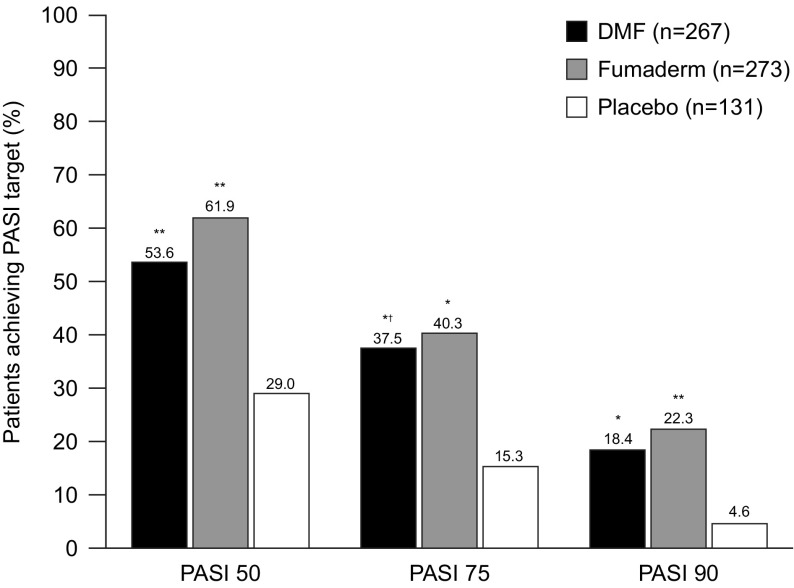
Percentages of patients achieving ≥ 75% improvement in Psoriasis Area and Severity Index (PASI 75; primary endpoint) at week 16 in the head-to-head DMF/FAE mixture comparator study [32]. Results are also shown for the secondary endpoints of 50% improvement (PASI 50) and 90% improvement (PASI 90). *p < 0.001 vs. placebo; **p < 0.0001 vs. placebo; ^†^p < 0.001 for noninferiority vs. Fumaderm. (Adapted from Mrowietz et al. [32])

In total, 839 patients were screened and 704 were randomized from 57 study sites (Table 3). Rates of treatment discontinuation were similar in the two active treatment arms (37.1% for DMF and 38.5% for the FAE mixture). The most common reasons for withdrawal were AEs with active treatment (23 and 24%, respectively) and lack of efficacy with placebo (15%). Significantly more patients achieved PASI 75 at week 16 with either DMF or the FAE mixture than with placebo, and DMF alone was non-inferior to the FAE mixture (37.5 vs. 40.3%; *p* < 0.001; Table 3; Fig. 1). Similar observations were reported for the secondary endpoints of PASI 50 and PASI 90 (Fig. 1). DMF was also non-inferior to the FAE mixture in terms of PGA 0–1 scores. Similar findings were reported for the secondary 3- and 8-week time points. BSA decreased from week 3 onwards in the DMF group, with significance vs. placebo being reported at week 8 and maintained at week 16 (Table 3). Rebound, defined as a worsening of PASI relative to baseline (PASI ≥ 125%) 2 months after the end of treatment, was very infrequent with both active treatments (1.1% with DMF vs. 2.2% with the FAE mixture and 9.3% with placebo). Mean rates of oral dose intake during the trial were very similar for DMF and the FAEs.

Treatment-emergent AEs were reported in 84% of patients in both active treatment groups, compared with 59.9% of patients receiving placebo (Table 3). Most events (approximately two-thirds in the DMF and the FAE mixture groups and half of those in the placebo group) were of mild severity. Gastrointestinal complaints were most frequent (Table 3) and included diarrhoea, abdominal pain, nausea and flatulence. Flushing was also reported in 18.3 and 16.3% of patients in the DMF and the FAE mixture groups, respectively. Similar incidences of lymphopenia were seen with both active treatments (Table 3).

Overall, the results from this study support a comparable clinical efficacy of DMF monotherapy to the empirical combination of ingredients in the approved FAE combination, when equivalent dosages of DMF are administered. These findings strongly support the assumption that DMF is the major active ingredient in FAE combination products.

### Clinical head-to-head comparisons of the FAE combination vs. biologics

There have also been some clinical trials comparing the efficacy of the FAE combination vs. biological agents used to treat psoriasis. A randomized, 24-week open-label trial investigated the efficacy of secukinumab compared with the licensed oral FAE mixture (Fumaderm®) in patients with moderate-to-severe psoriasis. Significantly more patients achieved PASI 75 in the secukinumab cohort, compared with the FAE cohort (*p* < 0.001). More patients also achieved a DLQI response of 0–1 with secukinumab, compared with FAEs (*p* < 0.001) [40]. Another randomized, open-label trial comparing the efficacy and safety of ixekizumab vs. FAEs and methotrexate in patients with moderate-to-severe psoriasis has recently been completed, but the results are not yet available [13].

## Conclusions

FAEs are well established in the management of psoriasis, although clinical trial evidence is limited until today. Nevertheless, FAE therapy has a long history of use and a favourable efficacy/safety profile in patients with moderate-to-severe plaque psoriasis. FAEs are used frequently in Germany and are available and used off-label in a number of other countries [6, 11, 12, 16, 33]. The significance of FAEs in the management of psoriasis is acknowledged by the Cochrane Collaboration [5] and by European and US official guidelines [3, 33, 34].

Accumulating pharmacological and clinical evidence has emphasized that DMF is the main active ingredient in FAE formulations (notably Fumaderm®). Most of its activity appears to be mediated via metabolic conversion to MMF. DMF in fact is a prodrug that, upon oral administration, is rapidly hydrolysed to MMF, the principal active molecule that elicits most of the anti-psoriatic effects [29].

Clinical trials have demonstrated that single DMF therapy is efficacious in patients with moderate-to-severe plaque psoriasis. A recent large-scale phase III randomized and placebo-controlled study has confirmed DMF as the clinically relevant ingredient of the FAE mixture [32]. The efficacy and safety of DMF when used as monotherapy is clinically equivalent to approved FAE mixtures. Therefore, the addition of MEF or other compounds is dispensable.
